# Clinical and epidemiological features of secondary glaucoma in a Mexican Tertiary Ophthalmology Hospital

**DOI:** 10.1007/s10792-025-03709-w

**Published:** 2025-08-13

**Authors:** Dalia Lozano-Arriaga, Susana Aguilar, Eduardo Izquierdo-Torres, Angel Zarain-Herzberg

**Affiliations:** 1 Department of Biochemistry, School of Medicine, National Autonomous University of Mexico, Mexico City, Mexico; 2 Mexican Social Security Institute (IMSS), Mexico City, Mexico

**Keywords:** Secondary glaucoma, Prevalence, Mexican population, Intraocular pressure

## Abstract

**Purpose:**

To determine the prevalence and types of secondary glaucoma in adult patients in a Mexican Tertiary Ophthalmology Hospital.

**Methods:**

Observational, cross-sectional, descriptive, and retrospective study in patients over 18 years of age diagnosed with secondary glaucoma in the period from January to June 2024. A non-probabilistic sampling was carried out within the established period, selecting patients with secondary glaucoma. The analysis included descriptive statistics of central tendency (mean, median, and mode) and dispersion (range, standard deviation) for numerical variables, as well as tables and/or graphs for categorical variables.

**Results:**

A total of 2390 records were analyzed, of which 987 corresponded to subsequent patients. A final sample of 1403 first-time patients was obtained, comprising 208 cases of secondary glaucoma (14.83%). The most frequent cause of secondary glaucoma was secondary to neovascularization (37.98%), in order of frequency, intraocular surgery (19.23%), corneal transplant (12.50%), ocular inflammation (10.10%), pseudoexfoliation syndrome (8.65%), ocular trauma (4.33%), pathologies associated with the lens (3.37%), other causes (1.44%), pigment dispersion syndrome and associated with corticosteroids (each 0.96%), finally associated with tumors (0.48%). The prevalence was 50% in women and 50% in men.

**Conclusion:**

Our results, although not representative of the entire population, offer valuable exploration for future research. Our database, as a national referral hospital for glaucoma cases, provides a robust and relevant source of data for understanding this condition. The findings of this study can be used to inform future research on secondary glaucoma and potentially improve diagnosis and treatment strategies for patients with this condition.

## Introduction

The term glaucoma refers to a progressive degeneration of the optic nerve associated with the loss of ganglion cells and thinning of the nerve fiber layer of the retina [1]. Secondary glaucoma is a group of eye diseases caused by ocular disorders or systemic factors that affect the drainage of aqueous humor, resulting in elevated intraocular pressure (IOP) and impaired visual function [2]. Common secondary ocular pathological processes include trauma, neovascularization, surgery- and intraocular lens-induced problems, medication-related problems, and tumors, among others.

In a study by Qian Liu et al. in China, one of the most extensive studies on secondary glaucoma prevalence was conducted at Henan Eye Hospital, the most common causes of secondary glaucoma were found to be Neovascularization 43.26%; Ocular Trauma 17.27%; Posterior Segment Surgery 16.75%; Lens Related Problems 7.60% and Inflammation 4.84% [3]. In a retrospective study by Gadia et al., where 2650 patients diagnosed as suspected glaucoma or glaucoma were analyzed, 21.84% of them had secondary glaucoma. Among the most common causes of secondary glaucoma identified in the study were post-vitrectomy (14%); closed ocular trauma (13%); corneal pathology (12%); aphakia (11%); neovascular (10%); pseudophakia (10%); steroid-induced (8%); uveitic (8%); and other causes (14%). In patients with secondary glaucoma, the male–female ratio was 2:2, and the age distribution was as follows: 25% were between 0 and 20 years old, 27% were between 21 and 40 years old, 30% were between 41 and 60 years old, and 18% were over 60 years old. The other categories included lens-induced glaucoma, post-penetrating keratoplasty glaucoma, tumor-related glaucoma, pseudoexfoliation syndrome, pigment dispersion glaucoma, glaucoma secondary to retinopathy of prematurity, aniridia, iridocorneal endothelial syndrome, and chemical injury [4].

In this study, we describe the clinical characteristics of secondary glaucoma in a Mexican tertiary medical center, with a focus on the most common secondary glaucoma, aiming to facilitate timely detection and treatment.

## Methods

This study was designed by the ethical principles for medical research and complies with the Declaration of Helsinki for research involving human subjects. It was approved by the Ethics and Research Committee of the Mexican Social Security Institute (IMSS) with institutional registration number R-2024-3502-167 in July 2024. The study subjects were identified from electronic medical records through an institutional electronic data warehouse. For the development of this study, an informed consent exception letter was made.

This is an observational, cross-sectional, descriptive, and retrospective study, conducted from January 1, 2024, to June 30, 2024, in the glaucoma department of the General Hospital of the National Medical Center La Raza, affiliated with the Mexican Social Security Institute, Mexico City. Non-probability sampling was performed due to the rarity and specificity of the condition studied and the strict inclusion criteria, which makes it difficult to access a broader population, thus to describe the characteristics of the selected sample of a specific population, all patients over 18 years of age with a diagnosis of secondary glaucoma were selected, defined by an IOP of 21 mmHg or more in at least one eye with or without treatment, associated with a primary pathology observable in the affected eye, with or without additional damage to the optic nerve or changes in the visual field [4]. The ocular pathological processes included in this study were secondary to trauma, lens-related problems, neovascularization, intraocular surgery, pseudoexfoliation syndrome, pigment dispersion syndrome, ocular inflammation, corticosteroid-induced, secondary to corneal transplant, tumors, and others. Adult patients with glaucoma diagnosed before the age of 18 were excluded. Records where IOP or a detailed ophthalmological examination that could not be documented were eliminated. The analysis included descriptive statistics of central tendency (mean, median, mode) and dispersion (range, standard deviation) for numerical variables, as well as tables and/or graphs for categorical variables. The prevalence of secondary glaucoma was explored stratified by sex and age. The data were processed using Microsoft Excel software, version 16.0, by Microsoft Corporation, 2019. The information was captured in tables and graphs.

## Results

A total of 2390 records were analyzed, comprising 987 records from patients with subsequent appointments and 1403 records from first-time patients. Of the 1403 records analyzed, 208 corresponded to patients (eyes) diagnosed with secondary glaucoma (14.83%), 773 to patients diagnosed with primary glaucoma (55.10%); in 422 patients, it was not possible to determine the type of glaucoma due to lack of information (30.08%).

Of the patients with secondary glaucoma, open-angle glaucoma was more frequent than angle closure glaucoma. Table 1 presents the prevalence of each subtype, along with the most frequent type of secondary glaucoma according to its classification. Regarding the most frequent cause of secondary glaucoma, it was determined that it was secondary to neovascularization; the rest are shown in Table 2.

**Table 1:** Frequency of patients according to glaucoma diagnosis

Diagnostic	Number of patients (n)	Percentage of patients (%)
Secondary Glaucoma	208	14.83
Open Angle	130 (n)	62.50
Most common type	Intraocular Surgery (n = 36), 27.7%	
Closed Angle	78 (n)	37.50
Most common type	Neovascularization (n = 46) 59%	
Total	208 (n)	100
Primary Glaucoma	773 (n)	55.10
Open Angle	530 (n)	68.56
Closed Angle	243 (n)	31.43
Total	773 (n)	100
No diagnosis due to incomplete record	422 (n)	30.08
Total	1403 (n)	100

**Table 2:** Frequency of patients diagnosed with secondary glaucoma according to their primary ocular pathology

Primary ocular pathology	Frequency (n)	Percentage (%)
Neovascularization	79	37.98
Intraocular surgery	40	19.23
Corneal transplantation	26	12.50
Ocular inflammation	21	10.10
Pseudoexfoliation syndrome	18	8.65
Ocular trauma	9	4.33
Lens-associated	7	3.37
Other	3	1.44
Corticosteroids	2	0.96
Pigment dispersion syndrome	2	0.96
Tumors	1	0.48
Overall	208	100

### Neovascular glaucoma

Of the 79 patients, the most frequent was secondary to proliferative diabetic retinopathy, with 70.88%, and 17.72% had vascular occlusions (central retinal vein occlusion and central retinal artery occlusion). On the other hand, glaucoma secondary to rhegmatogenous retinal detachment was detected in 3.79% of the patients. In 7.59% of cases, the cause could not be determined due to the chronicity and advanced stage of neovascular glaucoma. Of the 79 patients, 41.77% were classified as having open-angle (grade II) and 58.22% as having angle closure (grade III).

### Glaucoma secondary to intraocular surgery

Of the 40 patients with glaucoma secondary to intraocular surgery, 80% were due to vitreoretinal surgery. The vitreous substitute that most frequently caused glaucoma was silicone, followed by gas placement and cerclage. Other causes of intraocular surgery that led to secondary glaucoma included cataract phacoemulsification surgery with the placement of an intraocular lens (IOL) into the capsule, at 12.5%, followed by the placement of a lens in the anterior chamber and dislocation of a 1-piece intraocular lens to the anterior chamber, at 2.5%, respectively.

### Glaucoma secondary to corneal transplantation

All patients (100%) with glaucoma secondary to corneal transplantation were due to penetrating keratoplasty.

### Glaucoma secondary to ocular inflammation

In patients with glaucoma secondary to ocular inflammation, the diagnosis of non-granulomatous anterior uveitis (NGAU) was established in 90.47% of patients, 4.76% had unspecified chorioretinitis, and 4.76% had Posner-Schlossmann syndrome.

In NGAU patients, the cause could only be determined in 31.57% of patients, of which 31.57% were of infectious origin (Herpes and Toxoplasma) and 31.57% were of autoimmune origin (ankylosing spondylitis and relapsing polychondritis); the cause could not be determined for the remaining 68.42%.

### Glaucoma secondary to pseudoexfoliation syndrome

Eighteen patients were identified, with 66.66% being men and 33.33% being women. The mean age was 76.8 years for women and 75.5 years for men.

### Glaucoma secondary to ocular trauma

For closed ocular trauma, 66.66% was identified, while for open ocular trauma, 33.33% was identified. The male-to-female ratio was 2:1, and the mean age was 49 ± 13.8 years.

### Glaucoma secondary to lens pathology

85.71% of patients had phacomorphic glaucoma, and 14.28% had phacolytic glaucoma. The sample consisted of 42.85% men and 57.14% women, with a mean age of 59 ± 13.7 years.

### Glaucoma secondary to pigment dispersion syndrome and use of corticosteroids

In pigmentary glaucoma, there were two women aged 76 and 63 years, while in glaucoma associated with the use of corticosteroids, there were two men aged 78 and 44 years; the administration route was topical in both cases.

### Secondary glaucoma classified as “other”

The three identified causes were one case of ghost cell glaucoma and two congenital anterior segment defects that presented with glaucoma in adulthood.

### Glaucoma secondary to tumors

This form of glaucoma occurred in a 63-year-old patient diagnosed with functional pituitary adenoma, which was classified as secondary open-angle glaucoma due to endogenous cortisone.

### Age

**Fig. 1:**
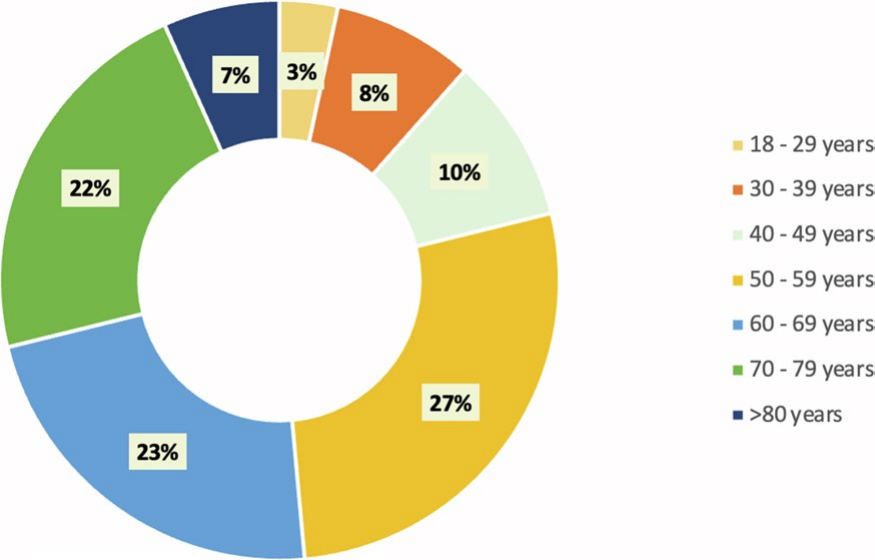
Frequency of patients with secondary glaucoma according to their age group

The mean age of patients diagnosed with secondary glaucoma was 59.8 ± 14.9 years, while patients diagnosed with primary glaucoma were on average 70.2 ± 10.2 years. Regarding age, it was decided to group them by decade of life, starting at 18 years of age. The most prevalent age group was patients between 50 and 59 years (27%). Figure 1 shows the prevalence found by age range.

Table 3 presents the distribution of patients with secondary glaucoma by age range, including the total frequency of cases, the frequency of secondary open-angle and angle-closure glaucoma, as well as the types of glaucoma in order of frequency within each age range.

**Table 3:** Frequency and types of secondary glaucoma by age group

Age range	No. of patients (n)	Open Angle n = (%)	Closed Angle n = (%)	Most common causes of secondary glaucoma (n)
18–29	7	4 (57.14%)	3 (42.35%)	1. Eye inflammation (n = 4) 2. Intraocular surgery, corneal transplant and eye trauma (n = 3)
30–39	17	13 (76.47%)	4 (23.52%)	1. Corneal transplant (n = 6) 2. Intraocular surgery (n = 4) 3. Neovascularization (n = 3) 4. Eye inflammation (n = 2) and Eye trauma (n = 2)
40–49	20	11 (55%)	9 (45%)	1. Eye inflammation (n = 6) 2. Corneal transplant (n = 4) 3. Lens pathology (n = 3) 4. Intraocular surgery (n = 2) and Neovascularization (n = 2) 5. Corticosteroid-induced, Other and Eye trauma (n = 3)
50–59	57	30 (52.63%)	27 (47.36%)	1. Neovascularization (n = 35) 2. Intraocular surgery (n = 9) 3. Corneal transplant (n = 7) 4. Eye inflammation (n = 3) and Eye trauma (n = 3)
60–69	47	34 (72.34%)	13 (27.65%)	1. Neovascularization (n = 20) 2. Intraocular surgery (n = 12) 3. Pseudoexfoliation syndrome (n = 4) 4. Eye inflammation (n = 2), Other (n = 2), Corneal transplant (n = 2) and Eye trauma (n = 2) 5. Pigment dispersion syndrome, Lens pathology and Tumors (n = 3)
70–79	46	29 (63.04%)	17 (36.95%)	1. Neovascularization (n = 14) 2. Intraocular surgery (n = 9) and Pseudoexfoliation syndrome (n = 9) 3. Corneal transplant (n = 6) 4. Eye inflammation (n = 3) and Lens pathology (n = 3) 5. Pigment dispersion syndrome and corticosteroid-induced (n = 2)
> 80	14	9 (64.28%)	5(35.71%)	1. Neovascularization (n = 5) and Pseudoexfoliation syndrome < n = 5) 2. Intraocular surgery (n = 3) 3. Eye inflammation (n = 1)
Total	n = 208	n = 130	n = 78	n = 208

### Sex

The sex distribution was found to be equal, with 104 patients of each gender, resulting in a prevalence of 50% for both males and females (Table 4). The prevalence of all types of secondary glaucoma stratified by sex is shown below (Table 5).

**Table 4:** Frequency of patients with secondary glaucoma according to their sex

Sex	No. of patients	Open Angle n = (%)	Closed Angle n = (%)
Female	104	n = 61 (58.65%)	n = 43 (41.34%)
Male	104	n = 69 (66.34%)	n = 35 (33.65%)

**Table 5:** Frequency of patients diagnosed with secondary glaucoma stratified by sex

Primary ocular pathology	Men		Women	
	Frequency (n)	Percentage (%)	Frequency (n)	Percentage (%)
Neovascularization	44	42.30	35	33.65
Corneal transplant	15	14.42	11	10.57
Intraocular surgery	13	12.5	27	25.96
Pseudoexfoliation syndrome	12	11.53	6	5.76
Eye inflammation	8	7.69	13	12.50
Eye trauma	6	5.76	3	2.88
Lens-associated	3	2.88	4	3.84
Corticosteroids	2	1.92	0	0
Other	1	0.96	2	1.92
Pigment dispersion syndrome	0	0	2	1.92
Tumors	0	0	1	0.96
Overall	104	100	104	100

### IOP

**Fig. 2:**
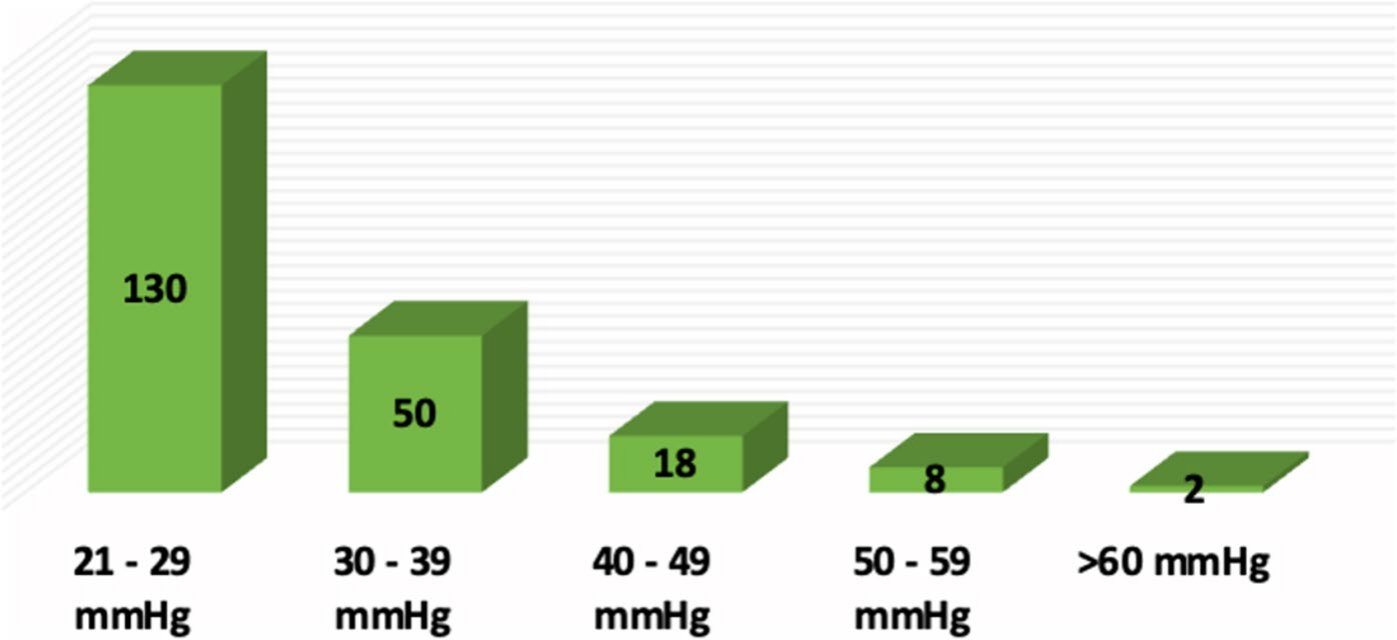
Distribution of patients diagnosed with secondary glaucoma according to Intraocular Pressure (IOP)

IOP was grouped by 10 mmHg, starting at 21 mmHg (Fig. 2). Patients with higher IOP (> 50 mmHg) were secondary to grade III neovascular glaucoma (90%), and only 1 due to intraocular lens dislocation into the anterior chamber. It is important to mention that many of these patients already had a history of topical hypotensive treatment or surgery.

## Discussion

The prevalence of secondary glaucoma in our analyzed population was 14.83%. The most frequent secondary glaucoma was neovascular, which represented 7.57% of all analyzed patients diagnosed with glaucoma. Diabetic retinopathy was identified as the most common cause of neovascular glaucoma, and the mean age of presentation was 61.49 ± 10.89 years.

According to the International Diabetes Federation (IDF), Mexico ranks seventh globally in terms of the number of individuals living with diabetes. Additionally, data from the National Institute of Public Health indicate that the prevalence of diabetes in Mexico is 18.3% [5]. Our results align with those found in the literature. A study conducted at a tertiary hospital in China, prepared by Na Liao et al., reported a prevalence of neovascular glaucoma of 5.8%. The mean age reported was 64.2 ± 14.0 years [6]. This highlights the significant public health challenge that Diabetes Mellitus poses on a global scale. Clinically, the course of neovascular glaucoma comprises three stages: (I) Pre-glaucoma or “rubeosis iridis” stage; (II) Open-angle glaucoma and (III) Angle-closure glaucoma [7]. In our study, the most frequent type of neovascular glaucoma was stage III or angle closure, with 59%, where even with surgical treatment, the outcome is torpid and usually ends in irreversible visual loss.

In glaucoma secondary to intraocular surgeries, the literature has reported elevated IOP mainly in vitreoretinal surgeries, including scleral buckle, pars plana vitrectomy, intravitreal silicone oil injection, gas injection, and steroid or anti-vascular endothelial growth factor (anti-VEGF) injections [8]. In our study, a prevalence of 19.23% of glaucoma secondary to intraocular surgery was reported, of which 80% were associated with vitreoretinal surgery, the most common cause being pars plana vitrectomy with silicone placement, the mean IOP was 28.1 ± 6.5 mmHg. A timely diagnosis is feasible since glaucoma in such scenarios is complicated by poor response to treatment, aggressiveness of the disease, previous manipulation of ocular structures that affect the success of filtering surgeries, as well as pathology of the retina and optic nerve that culminates in poor final visual results despite good surgical success, in addition to the fact that these patients are difficult to classify and quantify glaucomatous damage due to the alterations that exist at the same time in the retina and the technical difficulties in taking structural and functional studies.

In a study by Pohlmann et al., in Germany, where 270 patients diagnosed with anterior uveitis associated with viruses were analyzed, of which the most frequently found virus was Cytomegalovirus in 57 patients (21%), followed by Herpes simplex virus (HSV) in 77 patients (29%), HSV infection appeared mainly in elderly patients with a predominance of the female sex [9]. In our study, the most frequent cause of anterior uveitis was secondary to autoimmune diseases (19.04%) due to ankylosing spondylitis and relapsing polychondritis, and it was more frequent in women. Regarding the infectious cause, only two patients (9.52%) could be determined: one woman, aged 80 years, with a clinical diagnosis of Herpes simplex, and one man, aged 51 years, with a diagnosis of Toxoplasma. The remaining patients (71.4%) were still participating in the study protocol to define the etiology. When suspecting a case of ocular inflammation, it is always essential to initiate the study protocol as soon as possible, as more than half of our patients already have a chronic course of uveitis and have not yet received adequate causal treatment. The importance lies in the fact that, during this process, cataracts and secondary glaucoma developed that were difficult to control, which drastically compromised the patients' vision.

Glaucomatocyclitic crisis is a rare disease first described by Posner and Schlossman in 1948. It is classified as an inflammatory glaucoma [10]. In our study, a 70-year-old male patient was detected who presented a recurrent self-limiting inflammation since the age of 65. When he arrived at our center for the first time, the ophthalmologic examination revealed mild anterior chamber cellularity, discrete retrokeratic precipitates, open angle, absence of synechiae, associated with an IOP of 30 mmHg, for which the clinical diagnosis of Posner-Schlossman syndrome was established.

Glaucoma secondary to pseudoexfoliation syndrome is described in the literature as the most frequent secondary open-angle glaucoma [11]. In our population, it was found to be more frequent in men than in women, with a 2:1 ratio. In both groups, all individuals were over 60 years old, with a mean age of 75.8 ± 7.4 years. The mean IOP was 26.7 ± 7.4 mmHg. Its prevalence was not insignificant, occurring in 8.65%, which is why it is very important to always keep in mind that this syndrome not only entails ocular involvement, but also systemic associations [12].

All types of ocular trauma have the potential to cause elevated IOP in the affected eye through various mechanisms [13]. In a case series study by Bojikian et al., where they analyzed 515 eyes (patients) with a history of ocular trauma, they found that 23.3% of the patients developed elevated IOP. Of these, 6.2% of the patients in the same study developed glaucoma [14]. An incidence of glaucoma of 3.39% has been demonstrated after closed trauma to the eyeball at six months of follow-up after the injury [15], but ten years after the trauma, it is up to 10% [13]. In our study, a prevalence of 4.33% was found, of which 55.5% developed traumatic cataracts, being 2 times more common in men than women, with a 2:1 ratio, and in an active working age (mean age 49 years), which highlights the need for regular follow-up in this group of patients.

Some reports indicate that the incidence of lens-associated glaucoma is 2.4% at the time of presentation of senile cataracts, with a predominance in women [16]. In India, phacomorphic glaucoma is one of the most common causes of secondary angle closure glaucoma, with a reported incidence of 3.91% [17]. Multiple studies have shown that women outnumber men in both phacomorphic and phacolytic glaucoma [18, 19]. In our study, the prevalence of lens-induced glaucoma was 3.37%, the most common cause was phacomorphic glaucoma with 2.88%, and it was more common in women than in men.

Ghost cell glaucoma may appear after prolonged vitreous hemorrhage of various etiologies [20]. In our analysis, we found the case of a patient who presented with glaucoma secondary to ghost cells associated with vitreous hemorrhage secondary to long-standing proliferative retinopathy.

Glaucoma secondary to penetrating keratoplasty (PKP) is the second cause of graft failure. The incidence of glaucoma after PKP is relatively lower in the early postoperative period (9–31%) than in the late postoperative period (18–35%) [21]. In our study, a prevalence of 12.50% was detected, it was more common in men than in women, all patients were in the late postoperative period since.

In our study, a low prevalence percentage was found in glaucoma secondary to pigment dispersion syndrome (0.96%), the same percentage as that obtained in patients with glaucoma secondary to corticosteroid use. The topical presentation was used in two patients who were identified as responders to corticosteroids defined in current literature as an IOP above 21 mmHg and/or an increase of more than 5 mmHg compared to the initial value [22].

Only one patient was diagnosed with a functional pituitary adenoma that caused secondary open-angle glaucoma due to endogenous cortisone. Finally, 2 patients were detected with congenital anterior segment defects that presented with glaucoma in adulthood.

All of this reflects the great variety of diseases, not only at the ocular level but also at the systemic level, that can lead to the development of secondary glaucoma. Patients with this diagnosis tend to be younger, as the risk factors are mostly independent of age, unlike primary glaucoma, especially open-angle glaucoma, which is more a result of the aging process and genetic risk factors.

Regarding the limitations of this study, the following important points are highlighted: Our hospital receives many patients diagnosed with ocular trauma; we have a good record of short-term secondary glaucoma. However, our hospital does not provide medium- and long-term follow-up for this condition. Follow-up is carried out by local secondary hospitals, resulting in the loss of valuable information about their outcome.

The same applies to patients with glaucoma secondary to corneal transplants, as our Medical Center is a leading hospital in corneal transplants nationwide and has its own department, independent of the adult ophthalmology service, where the statistical analysis was conducted. In most cases, when a patient presents with secondary glaucoma, treatment is provided in that service. Only when the patient's condition is refractory to treatment and requires surgical intervention is the patient referred to our glaucoma department. Finally, a significant percentage of incomplete records (30%) were found, which could not be included in the study due to a lack of information regarding the ophthalmological examination, lack of patient follow-up, or inconsistencies in the diagnoses, leaving a window of diagnostic possibilities.

Currently, there is little information on the incidence of secondary glaucoma nationwide, as most attention focuses on primary glaucoma. Our results, although not representative of the entire population, offer valuable exploration for future research. Our database, as a national referral hospital for glaucoma cases, provides a robust and relevant source of data for understanding this condition. The findings of this study can be used to inform future research on secondary glaucoma and potentially improve diagnosis and treatment strategies for patients with this condition.

We believe that timely diagnosis is crucial due to the active working age of many patients, where vision loss can have a significant impact on quality of life and place a considerable burden on the healthcare system.

## Data Availability

No datasets were generated or analysed during the current study.

## Abbreviations

HSV: Herpes simplex virus
IDF: International Diabetes Federation
IMSS: Mexican Social Security Institute
IOL: Intraocular Lens
IOP: Intraocular Pressure
mmHg: Millimeter of mercury
NGAU: Non-Granulomatous Anterior Uveitis
PACG: Primary Angle Closure Glaucoma
PKP: Penetrating Keratoplasty
POAG: Primary Open-Angle Glaucoma
VEGF: Vascular Endothelial Growth Factor
